# The Effects of Hypoxia on the Immune–Metabolic Interplay in Liver Cancer

**DOI:** 10.3390/biom14081024

**Published:** 2024-08-17

**Authors:** Yubei He, Han Xu, Yu Liu, Stefan Kempa, Carolina Vechiatto, Robin Schmidt, Emine Yaren Yilmaz, Luisa Heidemann, Jörg Schnorr, Susanne Metzkow, Eyk Schellenberger, Akvile Häckel, Andreas Patzak, Dominik N. Müller, Lynn Jeanette Savic

**Affiliations:** 1 Department of Radiology, Campus Virchow-Klinikum, Charité-Universitätsmedizin Berlin, Corporate Member of Freie Universität Berlin and Humboldt Universität Zu Berlin, 13353 Berlin, Germany; yubei.he@charite.de (Y.H.); han.xu@charite.de (H.X.); yu.liu@charite.de (Y.L.); robin.schmidt@charite.de (R.S.); emine-yaren.yilmaz@charite.de (E.Y.Y.); luisa.heidemann@charite.de (L.H.); joerg.schnorr@charite.de (J.S.); susanne.metzkow@charite.de (S.M.); eyk.schellenberger@charite.de (E.S.); akvile.haeckel@charite.de (A.H.); 2 Experimental and Clinical Research Center, A Joint Cooperation of Max Delbrück Center for Molecular Medicine and Charité-Universitätsmedizin Berlin, 13125 Berlin, Germany; dominik.mueller@mdc-berlin.de; 3 Max Delbrück Center for Molecular Medicine in the Helmholtz Association, 10115 Berlin, Germany; stefan.kempa@mdc-berlin.de (S.K.); carolina.vechiatto@gmail.com (C.V.); 4 Institute of Translational Physiology, Charité-Universitätsmedizin Berlin, 10117 Berlin, Germany; andreas.patzak@charite.de; 5 Berlin Institute of Health at Charité-Universitätsmedizin Berlin, 10117 Berlin, Germany

**Keywords:** hepatocellular carcinoma (HCC), hypoxia, M2-like polarization, GC-MS-based metabolic profiling, 2-amino-butanoic acid (2A-BA)

## Abstract

M2-like macrophages promote tumor growth and cancer immune evasion. This study used an in vitro model to investigate how hypoxia and tumor metabolism affect macrophage polarization. Liver cancer cells (HepG2 and VX2) and macrophages (THP1) were cultured under hypoxic (0.1% O_2_) and normoxic (21% O_2_) conditions with varying glucose levels (2 g/L or 4.5 g/L). Viability assays and extracellular pH (pHe) measurements were conducted over 96 hours. Macrophages were exposed to the tumor-conditioned medium (TCM) from the cancer cells, and polarization was assessed using arginase and nitrite assays. GC-MS-based metabolic profiling quantified TCM meta-bolites and correlated them with M2 polarization. The results showed that pHe in TCMs decreased more under hypoxia than normoxia (*p* < 0.0001), independent of glucose levels. The arginase assay showed hypoxia significantly induced the M2 polarization of macrophages (control group: *p* = 0.0120,^0.1%^VX2-TCM group: *p* = 0.0149, ^0.1%^HepG2-TCM group: *p* < 0.0001, ^0.1%^VX2-TCM_HG_ group: *p* = 0.0001, and ^0.1%^HepG2-TCM_HG_ group: *p* < 0.0001). TCMs also induced M2 polarization under normoxic conditions, but the strongest M2 polarization occurred when both tumor cells and macrophages were incubated under hypoxia with high glucose levels. Metabolomics revealed that several metabolites, particularly lactate, were correlated with hypoxia and M2 polarization. Under normoxia, elevated 2-amino-butanoic acid (2A-BA) strongly correlated with M2 polarization. These findings suggest that targeting tumor hypoxia could mitigate immune evasion in liver tumors. Lactate drives acidity in hypoxic tumors, while 2A-BA could be a therapeutic target for overcoming immunosuppression in normoxic conditions.

## 1. Introduction

Hepatocellular carcinoma (HCC) is the most common primary liver cancer, the fifth most diagnosed cancer worldwide, and the third leading cause of cancer-related deaths [1]. Despite the variety of available treatments ranging from liver transplantation, resection, or locoregional therapies, to systemic treatments including immunotherapies, the prognosis of HCC remains unsatisfactory, with a survival of 36 months in early stages to only 6 months in advanced stages [2,3]. Unlike other cancer entities such as breast cancer, prostate cancer, or melanoma with representative targets for immune checkpoint inhibition [4,5,6], HCC appears to respond less well to single immunological treatments. While a variety of resistance mechanisms are under investigation, deregulated cellular metabolism plays a pivotal role in shaping an immune-inhibitory tumor microenvironment (TME) through lactate elevations and low pH in the proximal tumor surroundings [7]. This hallmark of cancer describes a hyperglycolytic metabolic tumor phenotype, which can lead to TME acidification due to increased glucose uptake and glycolysis as a result of the “Warburg effect” [8,9]. The TME acidification may be further exacerbated by hypoxia resulting from tumor proliferation that exceeds the tumor blood supply [10,11].

Specifically, tumor-associated macrophages (TAMs), one of the most abundant myeloid immune cells in the TME [12,13], are recruited to tumor tissue by chemokines, vascular endothelial growth factor (VEGF), and macrophage colony-stimulating factor (MC-SF) [14], that are upregulated under hypoxic conditions. Increasing evidence supports the important role of TAMs in carcinogenesis and cancer progression [15]. With the successful infiltration of TAMs into the tumor, TAMs can polarize to different subtypes, with M1-like and M2-like being the most popular ones [16]. Unlike pro-inflammatory M1-like macrophages, M2-like macrophages release anti-inflammatory cytokines such as IL-10 and arginase, which inhibit T-cell function and play an important role in orchestrating tumor growth and metastasis, as well as immune suppression and invasion of the TME [17]. The high plasticity of TAMs is reflected by dynamic changes in M1 and M2 polarization [18]. Therefore, exploring relevant factors in the regulation of macrophage polarization could potentially inform therapeutic decisions and improve the efficacy of immunomodulatory therapies.

Recent research showed that TAMs are preferentially found in hypoxic areas [19] that are also frequently observed in HCC [20]. Although the effects of hypoxia on cancers have been extensively studied, HCC is unique in that besides a hypoxic tumor microenvironment, the intentional induction of tumor hypoxia by the embolization of the tumor-feeding arteries is considered a guideline-approved treatment for HCC [2]. As the majority of patients are not amenable to curative therapies at diagnosis, intra-arterial therapies, particularly transarterial chemoembolization (TACE), are the most commonly used treatments for unresectable HCC worldwide [21], which are intended to induce tumor necrosis through ischemia and locally applied chemotherapy [22]. Nevertheless, the relatively high rates of incomplete response or local recurrence often require patients to receive multiple treatments, indicating the unmet clinical need to better understand the cross-talk of hypoxia, metabolism, and immunosuppression in the TME [23].

The objective of this study is to investigate the effects of hypoxia on tumor metabolism and macrophage polarization using a translational in vitro design and GC-MS-based metabolic profiling methods, including tumor-conditioned media (TCMs) and controlled oxygen supply to mimic different TME conditions.

## 2. Materials and Methods

### 2.1. Cell Culture

#### 2.1.1. Liver Tumor Cells

Two liver tumor cell lines were used in this experiment, a primary human hepatocellular carcinoma cell line HepG2 (ATCC, Manassas, VA, USA; no. HB-8065) and a metastatic sarcoma cell line VX2 (Sigma-Aldrich, St. Louis, MO, USA; no. 90030912), which is derived from the sarcoma of a female New Zealand White rabbit and commonly used in the translational rabbit liver cancer model [24]. Cells were cultured in RPMI 1640 supplemented with 10% fetal bovine serum (FBS) and 1% penicillin–streptomycin (Gibco, Grand Island, NY, USA), herein also referred to as the complete medium. Cells were grown under standard, normoxic cultivation conditions (37 °C, 5% CO_2_, 21% O_2_) and split once 70–80% confluence was achieved. In addition, subanalyses were performed in cells incubated under hypoxic culture conditions (37 °C, 5% CO_2_, 0.1% O_2_, Whitley H35 Hypoxystation, Don Whitley Scientific Limited company, Bingley, West Yorkshire, UK), or using a RPMI 1640 medium with additional glucose (final concentration of 4.5 g/L, herein referred to as the high-glucose complete medium). Further details and group designs will be explained in the following sections.

#### 2.1.2. Monocytes and Macrophages

Human acute monocytic leukemia cell line THP-1 (ATCC, Manassas, VA, USA; catalog no. TIB-202) was used as previously described [25]. Cells were cultured/subcultured in the complete medium and under standard cultivation conditions (37 °C, 5% CO_2_, 21% O_2_) to induce the differentiation of monocytes to macrophages; THP-1 monocytic cells (THP-Mo) were treated with 20 ng/mL phorbol 12-myristate 13-acetate (PMA, Sigma-Aldrich, St. Louis, MO, USA; no. P1585) for 24 h. The resulting macrophages were defined as ‘THP-MΦ cells’, which were used in all subsequent experiments [25].

### 2.2. Cell Viability Assay

Cells (THP-MΦ, HepG2, VX2) were seeded in 96-well transparent bottom polymer-based plates (3 × 10^3^ cells/well, 0.2 mL medium). Each cell type was divided into four groups with different medium or oxygen conditions, which were incubated as follows: (1) with complete RPMI 1640 medium (final concentration of glucose: 2 g/L) under normoxia (21% O_2_), (2) with complete RPMI 1640 medium (final concentration of glucose: 2 g/L) under hypoxia (0.1% O_2_), (3) with high-glucose complete RPMI 1640 medium (final concentration of glucose: 4.5 g/L) under normoxia (21% O_2_), and (4) with high-glucose complete RPMI 1640 medium (final concentration of glucose: 4.5 g/L) under hypoxia (0.1% O_2_). Cell viability was analyzed at 24, 48, 72, and 96 h of incubation by quantifying intracellular ATP levels using a luminescence-based kit (CellTiter-Glo, Promega, Fitchburg, WI, USA), following the manufacturer’s protocol.

### 2.3. Analysis of the Tumor Microenvironment (TME)

#### 2.3.1. Extracellular pH (pHe)

Cell lines (THP-MΦ, HepG2, VX2, 1 × 10^6^ cells/25 cm^2^ flask, 5 mL medium) were incubated under the different conditions as explained above (see Section 2.1.1). As a surrogate marker of tumor metabolism, and particularly, glycolysis resulting in lactate accumulation, the pHe of the medium was measured every 24 h from 0 to 96 h.

#### 2.3.2. Collection of Tumor-Conditioned Medium

Primary (HepG2) and metastatic liver cancer (VX2) cell lines were incubated under hypoxia (0.1% O_2_) with different concentrations of glucose (2 vs. 4.5 g/L) for 48 h, respectively. The medium was collected and filtered (1 × 10^6^ cells/25 cm^2^ flask, 5 mL medium) and is herein referred to as the tumor-conditioned media (TCM).

Accordingly, four different types of TCMwere harvested in this study:

1. Liver cancer cells (HepG2 and VX2) were incubated under hypoxia (0.1% O_2_) with complete medium (final concentration of glucose: 2 g/L) for 48 h, referred to as ^0.1%^HepG2-TCM and ^0.1%^VX2-TCM.
2. Liver cancer cells (HepG2 and VX2) were incubated under hypoxia (0.1% O_2_) with high-glucose complete medium (final concentration of glucose: 4.5 g/L) for 48 h, referred to as ^0.1%^HepG2-TCM_HG_ and ^0.1%^VX2-TCM_HG_.
3. Liver cancer cells (HepG2 and VX2) were incubated under normoxia (21% O_2_) with complete RPMI 1640 medium (final concentration of glucose: 2 g/L) for 48 h, referred to as ^21%^HepG2-TCM and ^21%^VX2-TCM.
4. Liver cancer cells (HepG2 and VX2) were incubated under normoxia (21% O_2_) with high-glucose complete medium (final concentration of glucose: 4.5 g/L) for 48 h, referred to as ^21%^HepG2-TCM_HG_ and ^21%^VX2-TCM_HG_.

**Figure 1:**
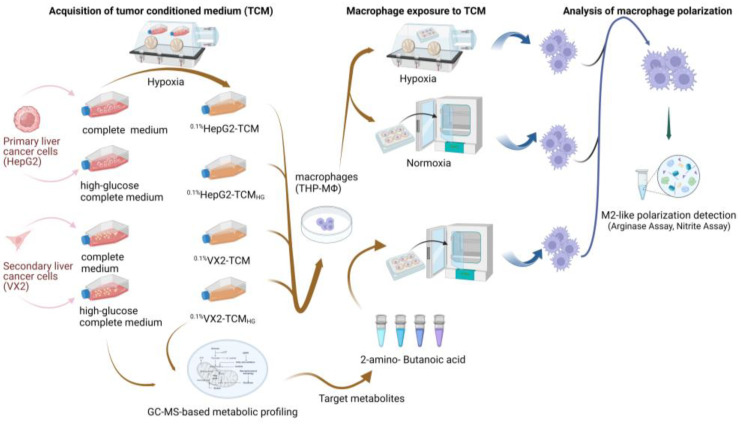
Flowchart summarizing the study design.

A flowchart summarizing the study design is depicted in Figure 1.

#### 2.3.3. GC-MS-Based Metabolic Profiling of TCM

All types of TCMs (see Section 2.3.2) were collected and filtered for GC-MS-based metabolic profiling. A total of 20 µL of cell medium was extracted in chloroform/methanol/water (2/5/1) and processed as previously described [26]. Samples were measured on a Pegasus ToF 4D, data were analyzed, and metabolites were identified as described in Opialla et al. [27]. Metabolites were quantified using 8-point calibration curves using an established quantification strategy [26]. Lactate was reported as a peak area and metabolites were evaluated in correlation matrices (see statistics below).

### 2.4. Effects of TCMs on the Polarization of Macrophages

#### 2.4.1. Arginase Assay

THP-MΦ cells (1 × 10^6^ cells/well in a 6-well plate, 2.5 mL medium) were cultured with complete medium (control) and different TCM (^0.1%^HepG2-TCM, ^0.1%^VX2-TCM, ^0.1%^HepG2-TCM_HG_, and ^0.1%^VX2-TCM_HG_) under normoxia (21% O_2_) and hypoxia (0.1% O_2_), respectively, for 48 h. Subsequently, the expression of arginase as an indicator of M2-like macrophage polarization in THP-MΦ cells was analyzed using an Arginase Activity Assay Kit (Sigma-Aldrich, Catalog No. MAK112, St. Louis, MO, USA) according to the manufacturer’s instructions.

#### 2.4.2. Nitrite Assay

THP-MΦ cells (1 × 10^6^ cells/well in a 6-well plate, 2.5 mL medium) were incubated under the same conditions as described in Section 2.4.1. The expression of nitric oxide (NO) in the THP-MΦ cells was detected by a Nitrite/Nitrate Assay Kit (Sigma-Aldrich, Catalog No. 23479, St. Louis, MO, USA) following the manufacturer’s protocol.

### 2.5. Effects of 2-Amino-Butanoic Acid (2A-BA) on the Polarization of Macrophages

#### 2.5.1. Cell Viability Assay

Following the findings of the metabolomics analysis, 2-amino-butanoic acid (2A-BA) was selected for further investigation into the effects of metabolites on macrophage polarization. THP-MΦ cells were treated with increasing concentrations of 2A-BA (0, 100, 200, 400 μM) under normoxic (21% O_2_) and hypoxic (0.1% O_2_) conditions for 24 h and 48 h, respectively. The luminescence-based assay was performed as described in Section 2.2.

#### 2.5.2. Arginase Assay

THP-MΦ cells (1 × 10^6^ cells/well in a 6-well plate, 2.5 mL medium) were incubated with different concentrations of 2A-BA (0, 100, 200, 400 μM) under normoxia (21% O_2_) for 48 h and analyzed using an Arginase Activity Assay Kit as described in Section 2.4.1.

### 2.6. Statistical Analysis

Experiments were repeated at least three times to ensure reproducibility. Data were tested for normal distribution and evaluated using two-way or three-way ANOVA with Tukey post hoc testing (>two groups). Data that did not fit the normal distribution were analyzed using the Kruskal–Wallis test. Statistical analysis was performed using Prism (Version 9.5, GraphPad, San Diego, CA, USA); *p* < 0.05 was considered statistically significant. In addition, the GC-MS-based metabolic profiling, data handling, and calculation were performed using R and in-house scripts. Quantitative metabolite data from GC-MS-based metabolic profiling were combined with supplemented glucose levels, growth conditions, as well as M2 polarization. For the metabolism part, the resulting data matrix was Z-transformed, and a Spearman correlation was performed. 

## 3. Results

### 3.1. Cell Viability Assay

**Figure 2:**
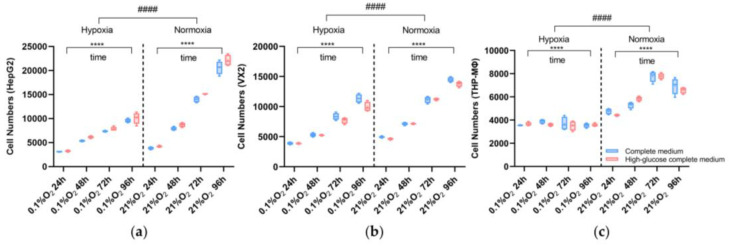
Viability assay for liver cancer cells (HepG2, VX2) and macrophages (THP-MΦ) incubated under normoxia or hypoxia with complete medium (blue box) or high-glucose complete medium (red box); all cell lines share the same legend. (**a**) HepG2 cell (**b**) VX2, and (**c**) THP-MΦ proliferation is displayed over 96 h. Three-way ANOVA was used for this analysis. ****, *p* < 0.0001 represents comparisons of the cell number changes over time within the groups (normoxic or hypoxic conditions) and ####, *p* < 0.0001 represents comparisons of the two groups for normoxia and hypoxia.

To investigate the impact of different microenvironments on cell proliferation, viability assays (Figure 2) were performed on cells exposed to varying oxygen and nutrient supply to mimic physiological tumor hypoxia and the effects of embolization. In liver tumor cells, proliferation was slower under hypoxic conditions compared to normoxic conditions (*p* < 0.0001). HepG2 proliferated faster in the high-glucose complete medium as compared to the regular medium, both under normoxic and hypoxic conditions (2 g/L vs. 4.5 g/L: *p* < 0.0001). VX2 cells were affected by glucose supply less than HepG2 cells, but the results were still significant (2 g/L vs. 4.5 g/L: *p* = 0.0005). Macrophage (THP-MΦ) proliferation was also decreased under hypoxia compared to normoxia (*p* < 0.0001) and generally slower than tumor cell proliferation, and the number of cells began to decrease after 48 h under hypoxia and 72 h under normoxia, respectively. In addition, there was no difference between the two glucose concentrations of the medium on cell viability (2 g/L vs. 4.5 g/L: *p* = 0.4403).

### 3.2. Extracellular pH

**Figure 3:**
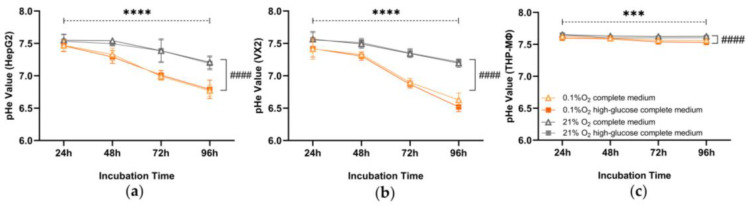
Longitudinal extracellular pH (pHe) measurements in the media of liver cancer cells (HepG2, VX2) and macrophages (THP-MΦ) incubated under normoxia (gray line) or hypoxia (orange line) with complete or high-glucose complete medium. (**a**) HepG2, VX2 (**b**), and THP-MΦ (**c**) keep the same design and share the legend. Three-way ANOVA was used for this analysis. ***, *p* < 0.001 and ****, *p* < 0.0001 are indicative of the difference between different time points; ####, *p* < 0.0001 is indicative of the difference between hypoxia and normoxia.

The pHe of the medium of both tumor cell lines remained largely steady under normoxic conditions, but gradually decreased over time in hypoxia indicating microenvironmental acidification (Figure 3). This difference caused by oxygen depletion was statistically significant (*p* < 0.0001), whereas different glucose concentrations did not affect pHe values significantly (HepG2, complete medium vs. high-glucose complete medium: *p* = 0.7014; VX2, complete medium vs. high-glucose complete medium: *p* = 0.5455). After 72 h in hypoxia, pHe decreased to acidic values less than seven. Compared to tumor cells, the pHe decrease in macrophages affected by hypoxia was less obvious but statistically significant (hypoxia vs. normoxia, *p* < 0.0001; complete medium vs. high-glucose complete medium, *p* = 0.0068).

### 3.3. Lactate Concentration of Liver Cancer Tumor Microenvironment (TME)

**Figure 4:**
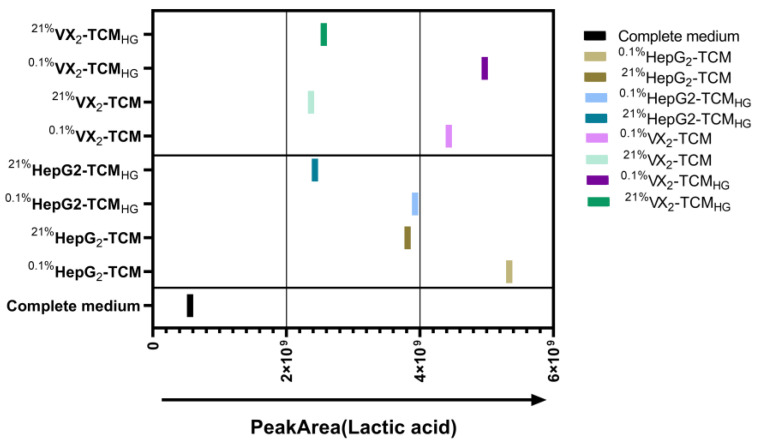
Peak area of lactic acid in the liver cancer tumor microenvironment (TME) based on GC-MS-based metabolic profiling. The left *Y*-axis shows the different types of tumor-conditioned mediums (TCMs). The direction of the arrow indicates an increase in peak area. Briefly, lactate concentrations were higher in the TCMs compared to the complete medium, and higher in the TCMs derived from hypoxic cancer cells compared to the TCMs derived from normoxic cancer cells, independent of glucose availability.

Compared to the high-glucose complete medium, the peak area of lactate (Figure 4) in the TCM was significantly and at least 2-fold higher, and the group with the highest increase exceeded 5-fold the original lactate peak. Additionally, the lactate peak area metabolic results showed increased lactate levels in the TCM of both liver cancer cell lines (VX2 cand HepG2) incubated under hypoxia as compared to normoxia. The lactate levels were comparable in the TCM from regular as compared to high-glucose complete medium in VX2, while ^0.1%^HepG2-TCM was a little higher as compared to ^0.1%^HepG2-TCM_HG_.

### 3.4. GC-MS-Based Metabolic Profiling of Liver Cancer Tumor Microenvironment (TME)

**Figure 5:**
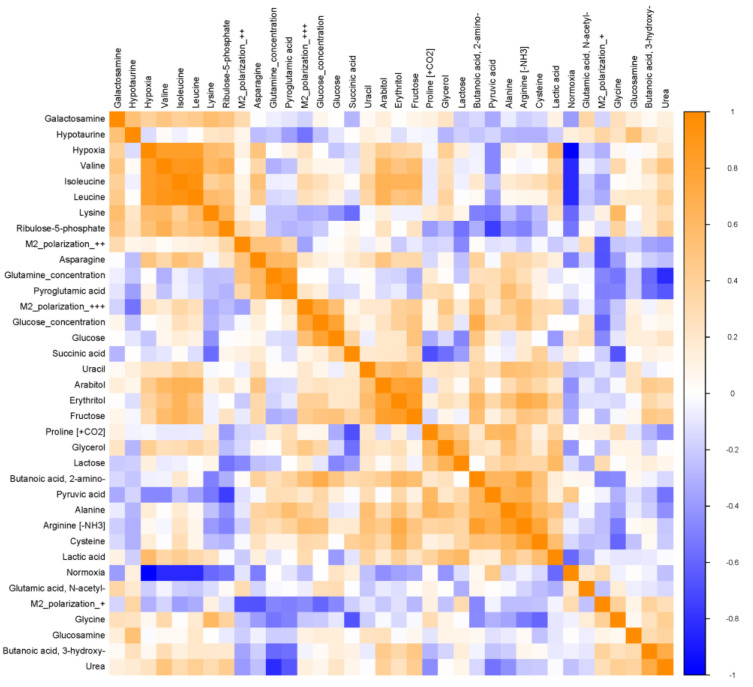
Heat map of the Spearman correlation matrix of the liver cancer tumor microenvironment (TME) GC-MS-based metabolic profiling. Two liver cancer cell lines were included in this cohort.

The heat map (Figure 5) shows the correlation of the major components of metabolites in TCMs collected from tumor cells cultured under different conditions with oxygen supply, glucose concentration, and M2 polarization. The correlation between each specific component is visible in the matrix. The progressively deeper orange color in the graph indicates an increasing correlation, and conversely, a darker blue color indicates a lower correlation. Specifically, when we focus on the metabolites associated with hypoxia, lysine, valine, leucine, isoleucine, asparagine, and lactic acid showed high positive correlation with hypoxia. When we focus on glucose concentration and metabolites in TCMs, the ones that have a noticeable positive correlation are as follows: isoleucine, erythritol, fructose, glycerol, pyruvic acid, Alanine, 2-amino-butanoic acid (2A-BA), Arginine [-NH3], and cysteine. Of all the metabolites, 2A-BA showed the strongest correlation with glucose concentration. It is also noteworthy that lactate was not correlated with glucose concentration. Subsequently, we focused on metabolites that were positively correlated with M2 polarization when the following metabolites increased with increasing M2 polarization (from “M2 polarization++” to “M2 polarization+++” in Figure 5) with an accompanying increase in correlation: valine, isoleucine, leucine, glucose concentration, 2-amino-butanoic acid (2A-BA), and cysteine. Among them, 2-amino-butanoic acid had the highest correlation with M2 polarization. In addition to the above metabolites, hypoxia and glucose concentration showed a tendency to affect M2 polarization more significantly.

### 3.5. Effects of Tumor-Conditioned Media (TCMs) on Macrophage Polarization

#### 3.5.1. Arginase Assay

**Figure 6:**
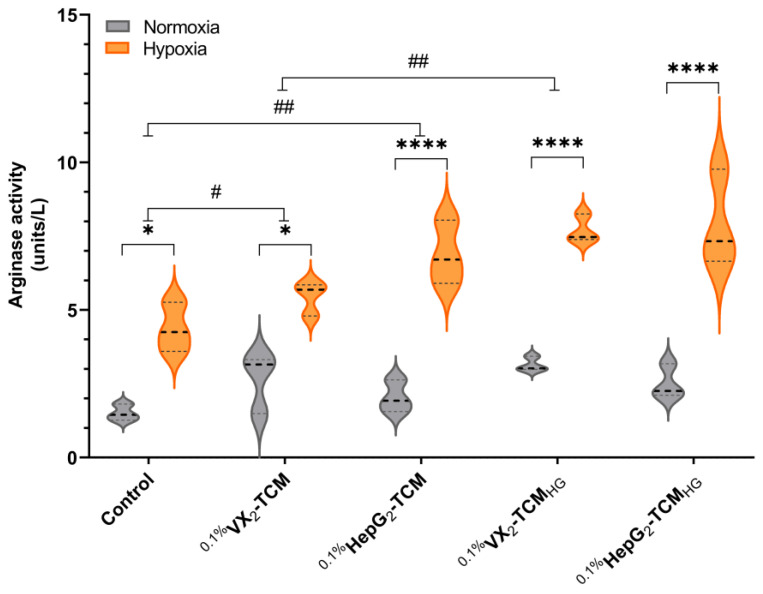
Violin plots showing arginase assay results for macrophages (THP-MΦ cells) incubated with different tumor-conditioned medium (TCM) types. The dotted line indicates the mean value. “Hypoxia (orange) or Normoxia (gray)” in the legend indicates if macrophages were cultured under 0.1% O_2_ (hypoxia) or 21% O_2_ (normoxia) in combination with different TCMs (see *X*-axis). Two-way ANOVA was used for this analysis. #, *p* < 0.05 and ##, *p* < 0.01: comparisons between two groups; *, *p* < 0.05 and ****, *p* < 0.0001: comparisons within a group.

Generally, arginase expression indicative of M2 polarization was significantly higher in macrophages incubated in hypoxia compared to normoxia in all five groups (Figure 6, control group: *p* = 0.0120, ^0.1%^VX2-TCM group: *p* = 0.0149, ^0.1%^HepG2-TCM group: *p* < 0.0001, ^0.1%^VX2-TCM_HG_ group: *p* < 0.0001, and ^0.1%^HepG2-TCM_HG_ group: *p* < 0.0001).

Additionally, arginase activity was distinctly increased when macrophages were cultured in TCMs compared to the normal complete medium (control group). This difference is amplified by the effects of hypoxic conditions (hypoxia: control group vs. ^0.1%^VX2-TCM group: *p* = 0.8389; control group vs. ^0.1%^HepG2-TCM group: *p* = 0.0356; control group vs. ^0.1%^VX2-TCM_HG_ group: *p* = 0.0026; and control group vs. ^0.1%^HepG2-TCM_HG_: *p* = 0.0013).

TCMs from tumor cells incubated with regular and high-glucose concentrations had similar effects on the polarization of macrophages when incubated in normoxia (normoxia: ^0.1%^VX2-TCM group vs. ^0.1%^VX2-TCM_HG_ group: *p* = 0.9989; ^0.1%^HepG2-TCM group vs. ^0.1%^HepG2-TCM_HG_ group: *p* = 0.9992). However, in macrophages incubated in hypoxia, arginase expression increased in the TCM_HG_ groups (hypoxia: ^0.1%^VX2-TCM group vs. ^0.1%^VX2-TCM_HG_ group: *p* = 0.0763, ^0.1%^HepG2-TCM group vs. ^0.1%^HepG2-TCM_HG_ group: *p* = 0.8671).

Overall, arginase expression tended to be higher when macrophages were in a hypoxic environment or in a conditioned medium where tumor cells had metabolized higher concentrations of glucose.

#### 3.5.2. Nitrite Assay

NO metabolite production, which is indicative of inflammation, was decreased in macrophages incubated with TCMs compared to the regular complete medium in both normoxia and hypoxia (21% O_2_: control group vs. ^0.1%^VX2-TCM group: *p* = 0.0066; control group vs. ^0.1%^HepG2-TCM group: *p* = 0.0031; 0.1% O_2_: control group vs. ^0.1%^VX2-TCM group: *p* = 0.0004; and control group vs. ^0.1%^HepG2-TCM group: *p* = 0.0014). In addition, among groups with the same medium, hypoxia induced higher NO levels than normoxia (hypoxia vs. normoxia: *p* = 0.0040; Figure S1).

### 3.6. Effects of 2-Amino-Butanoic Acid (2A-BA) on Macrophage Polarization

As GC-MS-based metabolic profiling revealed 2A-BA to be associated with M2 polarization of macrophages under normoxia, the effects of 2A-BA on macrophage viability and polarization were further explored.

#### 3.6.1. Cell Viability Assay of 2-Amino-Butanoic Acid (2A-BA)

**Figure 7:**
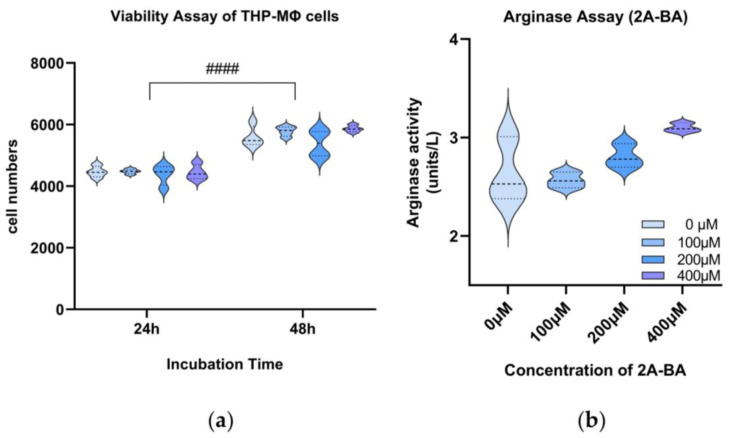
(**a**) Violin plots showing viability assay results for macrophages (THP-MΦ) incubated with increasing concentrations of 2-amino-butanoic acid (2A-BA) for 24 and 48 h. Two-way ANOVA was used for this analysis. ####, *p* < 0.0001: comparisons between two incubation time groups (24 h vs. 48 h). (**b**) Arginase assay for macrophages (THP-MΦ) incubated with increasing concentrations of 2-amino-butanoic acid (2A-BA). Kruskal–Wallis test was used for this analysis. *p* = 0.02: comparisons within a group of different concentrations of 2A-BA. (**a**,**b**) Plots share a legend and the gradual deepening of the blue color represents a higher concentration of 2A-BA.

Firstly, different concentrations of 2A-BA (<400 μM) did not affect the proliferation of macrophages when incubated under normoxia (Figure 7a), (*p* = 0.1775). As time increased, a statistical difference (*p* < 0.0001) was observed in the cell number of THP-MΦ. Cells increased in value generally under these conditions. This indicated that the cells were under good and stable proliferation under this culture condition. Therefore, 2A-BA can be used safely for the following experiments in the tested concentrations to explore its effect on macrophage polarization.

#### 3.6.2. Arginase Assay of 2-Amino-Butanoic Acid (2A-BA)

Increasing concentrations of 2A-BA were linked to higher arginase expression in macrophages and showed a statistical difference under normoxic conditions (*p* < 0.05), indicating M2 polarization (Figure 7b). These findings align with the GC-MS-based metabolic profiling, which showed that elevated 2A-BA levels were associated with M2 polarization.

## 4. Discussion

This study investigated the impact of hypoxia and the liver tumor microenvironment on the immuno-metabolic cross-talk in macrophages. The main finding of this study was that hypoxic conditions induced M2 polarization of macrophages compared to normoxic conditions, as confirmed by arginase assays and metabolomics. Furthermore, TCMs derived from both primary and secondary liver cancer cell lines induced M2 polarization under normoxic conditions independent of the glucose supply. M2 polarization was further promoted when both the tumor cells and macrophages were incubated with TCMs under hypoxia. The strongest induction of M2 polarization was observed under hypoxic conditions when the TCM was derived from tumor cells that had been supplemented with additional glucose.

Hypoxia is one of the key players in shaping immunosuppression in the TME [28]. It has been shown that T cells are deficient in the hypoxic regions of tumor tissues, and TAMs preferentially infiltrate in hypoxic regions with an increased probability of immune escape [29,30]. Recent studies further suggest that the infiltration of M2-like macrophages into the invasive front of a tumor may correspond to a lower overall survival rate [12,14,16].

Macrophages can switch from an oxidative-phosphorylation-based aerobic mode to a glycolysis-based anaerobic mode or vice versa [31]. Most studies indicated that glycolysis is a key metabolic event in M1 macrophages, especially under hypoxia, which may explain the increasing NO expression. On the contrary, the role of glycolysis in the M2 macrophages is highly debated. It has been shown that glycolysis is active in M2 cells, and that the blockade of glycolysis using 2-DG may inhibit M2 polarization and the macrophage function [32,33]. However, studies also showed that glycolysis is not required for M2 differentiation as long as OXPHOS remains complete [34]. This suggests a more flexible metabolic activity of M2 macrophages as compared to M1. Finally, this metabolic plasticity allows macrophages to accommodate the lack of oxygen, like M1, but also induces the expression of M2-like markers that promote tumor growth.

This study further suggests that hypoxia may not only induce the polarization of macrophages directly, but it can also alter tumor metabolism, which in turn also affects macrophage polarization in the TME. GC-MS-based metabolic profiling of the TCM demonstrated increased lactate levels under hypoxia, which in turn also correlated with M2 polarization.

GC-MS-based metabolic profiling is uniquely advantageous for the characterization of the microenvironment as it detects and quantifies metabolites and allows for exploring the relationship between different metabolites and predefined outcomes using metabolic correlation matrices. Firstly, lactic acid was peaking in the TCM derived from tumor cells that were incubated under hypoxia as compared to the normoxic TCM or regular culture medium. Interestingly, lactate levels in the TCM did not correlate with glucose supply to the tumor cells [35]. Accordingly, the pHe in the TCM decreased over time under normoxic conditions but even more under hypoxic conditions but was independent of the glucose supply. There is vast evidence to support that low pHe following lactate accumulation contributes to the immunosuppressive environment, further through the quiescence of the cellular antitumor immune response, promoting immune evasion by cancer cells [36,37,38]. Of course, since there are various substrate and product shifts among metabolites, these changes are rather relative than reflective of an absolute transformation. At the same time, the drivers of such complex processes are never monolithic, which is why we introduced the GC-MS-based metabolic profiling approach, aiming at full-scale knowledge. Therefore, the correlation matrix seemed to be appropriate to visualize the effects of metabolites identified using GC-MS-based metabolic profiling.

Despite the decreasing pHe, tumor cells maintained a constant proliferative rate under hypoxia, underscoring their adaptability to survive in hostile microenvironments that would typically inhibit normal cell growth. This cancer trait is majorly driven by “metabolic plasticity”, which enables tumor cells to adapt their metabolism in response to environmental changes, allowing for tumor invasion and metastasis in the face of nutrient scarcity, oxygen deprivation, and other stressors [39]. On the contrary, macrophage proliferation stagnated after 48 h of hypoxia or 72 h of normoxia, while the pHe of the culture medium remained stable over time under normoxic and hypoxic conditions. However, under hypoxia, the pHe was generally lower than under normoxic conditions, possibly because macrophages, just like most normal cells, can switch to anaerobic glycolysis for temporary energy supply when oxygen is depleted [40].

In the TCM obtained from normoxic conditions, elevated concentrations of 2A-BA were detectable, demonstrating a strong correlation with the M2 polarization of macrophages. 2A-BA, also known as alpha-aminobutyric acid (AABA), is a nonproteinogenic amino acid generated as a by-product of either cysteine biosynthesis or the metabolic pathways of methionine, threonine, serine, and glycine [41,42]. Most research on the clinical role of 2A-BA that has focused on plasma level is associated with the progression of sepsis and high-grade glioma [41,43]. A recent study involved immune cells and their function; they claimed that 2A-BA inhibited the activation and pro-inflammatory function of M1 macrophages, thereby protecting mice from LPS and CLP-induced sepsis and DSS-induced inflammatory bowel disease [44], but the study did not discuss the effects of M2 macrophages. It has been reported that 2A-BA was found to be 8.2 ± 7.8 µmole/L (in red blood cells) and 19.9 ± 4.1 µmole/L (in plasma) in healthy humans but increased in advanced sarcoma patients [45,46]. In our study, increasing the concentration of 2A-BA up to 400 μM was proportionally associated with the M2 polarization of macrophages. Additionally, GC-MS-based metabolic profiling revealed a correlation between 2A-BA and M2 polarization. Overall, these findings suggest that 2A-BA drives metabolically mediated immunosuppression in normoxic liver tumors. Therefore, the inhibitors of 2A-BA could potentially be used to manipulate the tumor microenvironment in a favorable manner, treating cancers by reducing immunosuppression and making them more susceptible to immunotherapies.

In addition, the relationship between glucose and several metabolites became apparent in the metabolomic analyses. First, arginine was highly correlated with glucose concentration in the TCM. As a cosubstrate of two classical metabolic pathways (NOS and ARG), arginine can act as a competitive inhibitor of both pathways [47], thus affecting the dynamic homeostasis of the TME. Second, among all the metabolites, 2A-BA showed the strongest correlation with glucose concentration, and it also correlated with arginine and lactate, both of which have been shown to play a key role in tumor metabolism and macrophage polarization [48,49].

Arginase is a key biomarker of M2 activation. High arginase expression levels result in increased L-arginine metabolism, and the remaining metabolites will participate in and sustain regenerative processes, such as angiogenesis [50]. Arginase competes with iNOS for arginine, and increasing arginase expression will block NO production, which can inhibit M1 to M2 repolarization [51,52]. Tumor-licensed myeloid cells activate both ARG and NOS pathways [50]. Thus, we measured the expression variations of arginase and NO in macrophages cultured under different oxygen and nutrient conditions. We found that the incubation of macrophages with the TCM obtained from hypoxic tumor cells, particularly, if incubated with a higher glucose supply, induced increased arginase expression indicative of M2 polarization. These findings are in accordance with previous studies that reported on elevated arginase expression in macrophages incubated with TCMs.

Regarding the effects of glucose availability on tumor metabolism, abundant glucose may be metabolized through aerobic glycolysis (Warburg effect) and is associated with the release of more tumor-associated cytokines and exosomes, which further enhance the M2 polarization of macrophages [53]. However, in this study, glucose supply may have exceeded the saturation limits of tumor cells and the remaining excess glucose in the TCM may have directly impacted the macrophages in the subsequent experiments. It has been reported that single-cell glucose consumption was significantly higher in tumor myeloid cells than in tumor-infiltrating T cells or cancer cells [54], but the effect of glucose on their polarization remains unknown.

Accordingly, the NO production results show that macrophages cultured with TCMs showed a significant decrease in NO production. There is substantial evidence demonstrating the dual role of NO in tumorigenesis, with high levels of NO leading to oxidative stress and nitrosation, DNA damage, and apoptosis. In contrast, low levels of NO have an antiapoptotic effect that stimulates angiogenesis and promotes tumorigenesis [55]. At the same time, NO can inhibit the conversion of M1 to M2, and a decrease in NO may cause a certain degree of decrease in immune monitoring [31], resulting in the partial polarization of M1 to M2.

HCC has been described as a highly acidified and hypoxic tumor previously, and the metabolic reprogramming caused by hypoxia is hypothesized to contribute to tumoral immune evasion [56]. Several therapeutic approaches have been introduced to target glycolytic enzymes in HCC. However, only a few of them have made it to clinical translation, and none of them have entered guideline recommendations yet [57,58]. Considering the evolution of immunotherapies and their emerging role in oncology, including advanced HCC, metabolic targeting may enter the scientific limelight again to modulate the immune TME, converting “cold tumors” into rather “hot tumors” [59], that are potentially more susceptible to immunostimulating therapies. Additionally, such targeted therapies may aid the efficacy of TACE by preventing supposedly negative ischemia-induced immunosuppression in the TME.

This study has some limitations. First, the in vitro study design may not fully reflect the complex and variable in vivo TME, although we designed the experiments to mimic the microenvironment of liver tumors as much as possible using multiple variations of TCMs. Therefore, we still need to further explore and prove the above findings in living organisms. Secondly, we only verified M2 polarization from a metabolic point of view. The next studies may incorporate further immunologic, cellular phenotypic, and other assays. Thirdly, tumor metabolism and macrophages may also be affected by cytokines, exosomes, and extracellular matrix components, but this study focused only on metabolites in the TCM and oxygen supply. For this proof-of-concept study, two liver cancer cell lines were used, one of which was a primary human HCC and the other one was a metastatic rabbit sarcoma cell line. However, both cell lines are frequently studied and have been shown to exhibit different metastatic potential as well as pHe [24]. Specifically, VX2 cells are frequently used in the rabbit liver tumor model as the tumors develop imaging characteristics, hypervascularity, central necrosis, and low pHe that are comparable to proliferative HCC [56].

## 5. Conclusions

In conclusion, this study revealed the impact of oxygen on macrophages and metabolically mediated immunosuppression in liver tumors, suggesting that targeting tumor hypoxia may promote immune permissiveness of the TME. Besides lactate as a driver of acidity in hypoxic tumors, metabolomic analyses identified 2-amino-butanoic acid as a potential player promoting M2 polarization in normoxic environments. The experiments are sought to mimic variations of the inherently heterogenous TME and to improve our understanding of the immuno-metabolic cross-talk in liver cancers. The findings emphasize the need to better characterize tumors to help design new targeted therapeutic approaches and inform personalized treatments for patients with HCC based on their TME profile.

## Data Availability

The datasets used and/or analyzed during the current study are available from the corresponding author upon reasonable request.

## Supplementary Materials

The following supporting information can be downloaded at: https://www.mdpi.com/article/10.3390/biom14081024/s1. Figure S1: nitrite assay. Figure S2: qPCR to evaluate the potential of HepG2 TCMs under normoxia and hypoxia for M2 macrophage polarization. Figure S3: qPCR to evaluate the potential of 2-amino-butyric acid (2A-BA) under normoxia for M2 macrophage polarization.
