# Supplementary material for: The Effects of Hypoxia on the Immune–Metabolic Interplay in Liver Cancer
Source: Biomolecules. 2024 Aug 17;14(8):1024. doi: 10.3390/biom14081024 (PMC11352590; doi:10.3390/biom14081024)
Supplement: Supplementary file 1 [file biomolecules-14-01024-s001.zip › biomolecules-3082484-supplementary.pdf]

Supplementary materials and methods

RT-qPCR to evaluate the potential of HepG2 TCMS and 2A-BA for M2 macrophage polarization.

THP-MΦ cells were incubated under the same conditions as described in 2.4.1 (only used the $^{0.1\%}$HepG2-TCM and $^{0.1\%}$HepG2-TCM$_{HG}$ group) and 2.5.2. The total RNA of cells was extracted with TRIzol reagent (ABP Biosciences, #FP312A), and was quantified by Nanodrop 2000 spectrophotometer (Thermo Fisher). mRNA contained in 500 μg total RNA was reverse transcribed using a random primer (Thermo, #48190-011), dNTP Mix (10 mM each, Thermo Fisher, #R0192), and SuperScript™ II Reverse Transcriptase kit (Invitrogen, #18064014). Amplification reaction assays contained power up SYBR Green Master Mix (Thermo Fisher, #A25776), and primers were synthesized by TIB Molbiol Syntheselabor GmbH (Berlin, Germany). The sequences of the primers are as follows. CD206: (F)TTCCTTTGGACGGATGGACG (R)TAAACTGCTTGAAGCGGCCA. IL-10: (F) GGTTGCCAAGCCTTGTCTGAG; (R) GATGACAGCGCCGTAGCC. GADPH: (F) GCACCGTCAAGGCTGAGAAC; (R)TGGTGAAGACGCCAGTGGA. GAPDH was used as the reference gene. The mRNA abundance was quantified in RQuantStudio™ 3 Real-Time PCR System (Applied Biosystems™, #A28572), according to the manufacturer's instructions.

Results

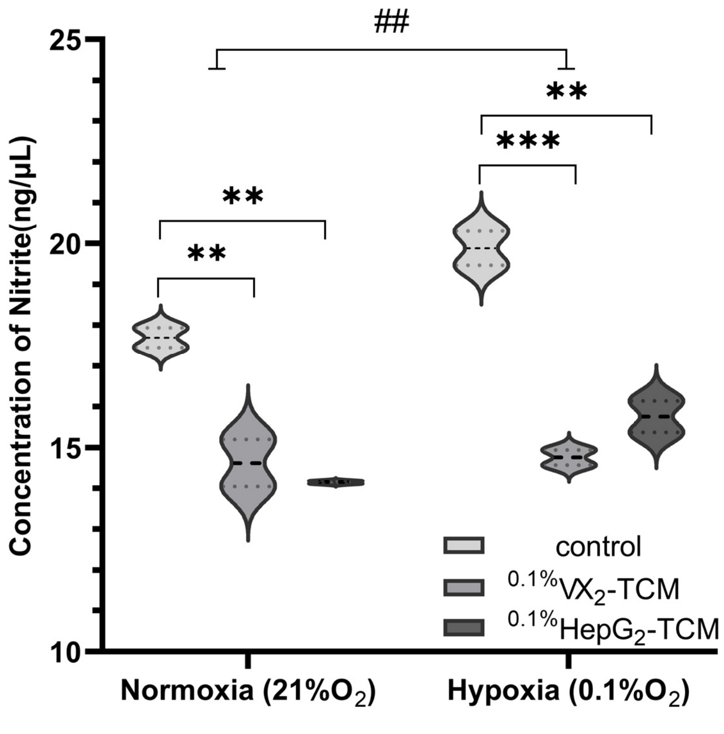

Figure S1: nitrite assay

1. Figure S2 investigated the impact on M2 macrophage polarization indicated by CD206 and IL10 overexpression by $^{0.1\%}$HepG2-TCM and $^{0.1\%}$HepG2-TCM$_{HG}$ group under normoxic and hypoxic conditions. Given that qPCR is a semi-quantitative experiment, the $^{0.1\%}$HepG2-TCM group served as a control for this comparison. It showed that higher glucose TCM induced higher M2 polarization-related mRNA expression compared to regular glucose.

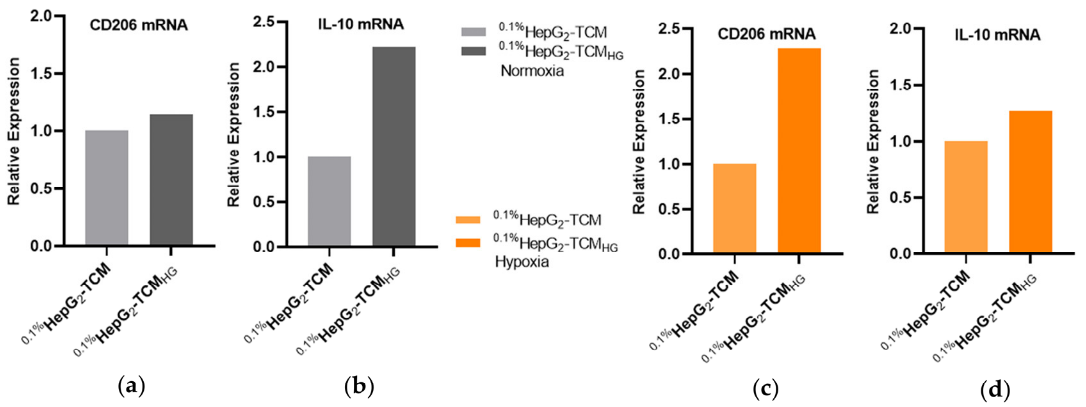

Figure S2. qPCR to evaluate the potential of tumor-conditioned medium from HepG2 cancer cells incubated with regular and high glucose levels under normoxia and hypoxia for M2 macrophage polarization. The box plots show the relative expression of CD206 (a, c) and IL-10 (b, d) indicative of M2 polarization. Macrophages (THP-MΦ cells) were incubated with $^{0.1\%}$HepG2-TCM and $^{0.1\%}$HepG2-TCM$_{HG}$ under normoxia (a, b) and hypoxia (c, d). Expression levels were normalized to 1 in the $^{0.1\%}$HepG2-TCM group used as a control in this comparison.

2. Figure S3 revealed the impact on M2 macrophage polarization indicated by CD206 and IL10 overexpression by different concentrations of 2A-BA under normoxic conditions. The 0μM group served as a control for this comparison. It showed that there was a dose-dependent association of 2A-BA with M2 polarization up to the concentration of 200μM under normoxia.

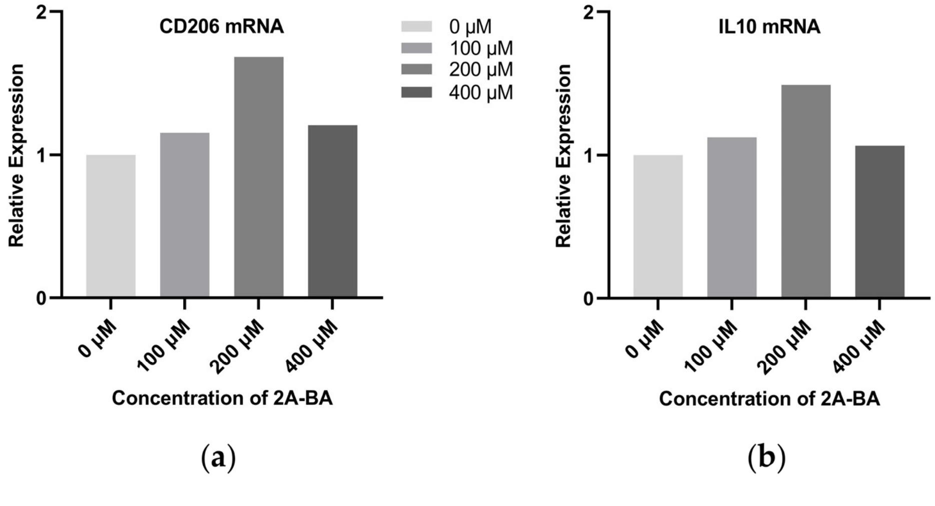

Figure S3. qPCR to evaluate the potential of different concentrations of 2-amino-butyric acid (2A-BA) under normoxia for M2 macrophage polarization. Relative (a) CD206 and (b) IL-10 mRNA expression are indicative of M2 polarization. Expression levels were normalized to 1 in the 0μM control group.
